# Beverage Consumption: Are Alcoholic and Sugary Drinks Tipping the Balance towards Overweight and Obesity?

**DOI:** 10.3390/nu7085304

**Published:** 2015-08-11

**Authors:** Sally D. Poppitt

**Affiliations:** Human Nutrition Unit, School of Biological Sciences, Department of Medicine, University of Auckland, Auckland 1024, New Zealand; and Riddet Institute, Palmerston North 4442, New Zealand; E-Mail: s.poppitt@auckland.ac.nz; Tel.: +64-9-373-7599; Fax: +64-9-630-5764

**Keywords:** alcoholic beverages, sugar-sweetened beverages, body weight, obesity

## Abstract

The role that energy-containing beverages may play in the development of overweight and obesity remains highly controversial, in particular the alcoholic and sugar-sweetened beverages (SSB). Both of these beverage formats have been increasing as a percentage of the westernized diet over the past 20 years, and both have contributed significantly to an increase in energy consumed in liquid form. Data from epidemiology and intervention studies however have long been contradictory, despite mechanistic evidence pointing towards poor compensation for addition of “liquid” energy from these two sources into the diet providing a strong rational for the balance to be tipped towards weight gain. Regulatory and government intervention has been increasing globally, particularly with respect to intake of SSBs in children. This narrative review presents evidence which both supports and refutes the link between alcohol and carbohydrate-containing liquids and the regulation of body weight, and investigates mechanisms which may underpin any relationship between increased beverage consumption and increased energy intake, body weight and adiposity.

## 1. Introduction

The causal role that beverages may play in the development of overweight and obesity has been highly controversial over the last 15 years, drawing into the debate the multiple and varied communities of researchers, clinicians and industry as well as national government. This narrative review aims to present recent evidence, both in support of and refuting the link, between energy-containing liquids that we drink, as consumed in many and various forms within our diet, and the effect that they may have on regulation of body weight. It aims also to investigate some of the mechanisms which may underpin any relationship between increased beverage consumption and increased energy intake, body weight and adiposity. Energy-containing beverages appear in wide ranging formats including alcoholic beverages (ABs), “soft” or “fizzy” beverages or fruit juices with added sugar (sugar sweetened beverages, SSBs), diet drinks, energy drinks, sports and isotonic drinks, and infusions such as coffee and tea. Newer formats such as energy drinks which first appeared on the market in the 1980s, have been extremely successful with more than 58 billion L sold globally [1], although still representing <1% of the global non-ABs market. The two nutritive beverage formats that are of particular interest and relevance to the control of body weight are the ABs and the SSBs, and which are the focus of this review. In the US alone they represent the two largest beverage categories with >9 billion (19% of beverage total including bottled water category) and >13 billion (27% of beverage total) gallons consumed respectively in 2012 [2] and, as energy-containing beverages, both present a risk for obesity [3,4]. Data sources for this review included the electronic databases PubMed and Medline, and incorporated recent systematic reviews and meta-analyses. Observational studies, randomised clinical trials (RCTs), and experimental data were included.

### 1.1. Alcoholic Beverages (ABs)

ABs have been consumed since the earliest days of human society, with the most ancient of fermented beverages being beer and wine with relatively low alcohol content [5]. The Arabs introduced distilling into Europe in the Middle Ages, where it was widely believed it to be the elixir of life and the remedy for many diseases, leading to the naming of spirits such as whisky in Gaelic as *usquebaugh*, the “water of life”. Gin was the original alcohol distilled from grain, produced from excess corn, and consumption of large quantities of alcohol which lead to adverse nutritional consequences of alcoholism in turn lead to The Gin Act of 1751 in the United Kingdom which imposed a duty on spirits to bring excessive alcohol consumption under control [6]. Even as recently as the late 1800s it was believed that alcohol was of great nutritional importance. Rum for example was developed as a by-product of sugar cane in the Caribbean and distributed to the British navy in the false belief that it would protect against scurvy [7]. In Westernised countries, alcohol is now consumed by the majority of the adult population, and in general, the greater the economic wealth of a country globally the more alcohol is consumed, with high-income countries having the highest alcohol per capita consumption [8]. Over recent decades total per capita intake of ABs has increased rapidly. For example, in the UK alcohol consumption doubled during the 40 years between 1960 and the millennium [9]. The average intake of those who drink alcohol is approximately 10–30 g/day, with an energy content of 290–880 kJ. This represents up to 9% of a typical individual’s total energy intake of 10 MJ [10]. It has been proposed that moderate alcohol consumers may add ABs into their daily energy intake rather than balancing the intake with a parallel decrease in solid foods, and thereby in turn driving a positive energy balance [11]. Certainly there has long been epidemiological evidence to support the premise that alcohol-derived energy added to food intake does not significantly decrease the average intake of the three other food macronutrients [12], and so would be predicted to promote weight gain. Alcohol has been identified as a component cause of >200 medical conditions of which liver cirrhosis, cancers and alcohol-related physical injury are most prevalent [8], and in 2012 ~5% of the global burden of disease and injury and ~6% of global deaths were attributable to alcohol consumption [8]. The role of alcohol consumption in overweight and obesity, recently recognised as a disease by the American Medical Association (AMA) [13], remains poorly understood but with a number of early studies suggesting a positive association such that consumption of ABs may be a risk factor for obesity [14,15].

### 1.2. Sugar Sweetened Beverages (SSBs)

Unlike ABs, the SSBs are commonly consumed by both adults and children, many of which represent global marketing brands. For many years intake of SSBs dramatically increased in many countries [16,17,18], an issue of particular concern in the United States where up to 50% of the population is estimated to purchase and consume a sugar-containing beverage on any given day. In 2010 this led to dietary guidelines being issued within the US recommending a limit on consumption of beverages with added sugars [19], which may in turn have helped to halt the rise in consumption [20], although SSBs remain the largest contributor to added sugar containing 20–40 g of sugar per serving [21,22,23]. Notably SSB consumption globally continues to rise as shown in a recent cross-national analysis of 75 countries [24], from 9.5 gallons (~36 L) per person per year in 1997 to 11.4 gallons (~43 L) in 2010. Again, as for ABs, the issue that is key is whether high SSB consumers add these energy-containing beverages into their diet (energy addition) rather than balancing intake with a parallel decrease in other food and/or beverage items (energy substitution/compensation). Without energy compensation, high intake of SSBs would be expected to drive a positive energy balance and so promote weight gain.

These nutritive sweeteners most commonly comprise sucrose (50% glucose, 50% fructose), high fructose corn syrup (HFCS, commonly a combination of glucose and fructose), or concentrates of fruit juice (fructose). The controversy as to whether there is a causal role between high consumption of energy-containing SSBs and high rates of weight gain and obesity has been rigorously debated over several decades. Support for an association between the two comes from large observational studies [4], but demonstrating causation, a step which arguably is required in order to provide sufficient strength of evidence to promote major national and international public policy initiatives, has been more difficult and the debate remains ongoing. Further controversy is leant to the topic through widespread discussion as to the role that the beverage industry may have played in building the scientific evidence [25] and whether this should be of concern to the scientific community [26,27]. Nevertheless, the evidence has been building. In 2014 WHO proposed a cut in their 2002 recommendation, which was to maintain intake of free sugars below 10 percent of total energy (10en%) and which had been shown to be well substantiated in a recent systematic review [28], with a more ambitious “ideal” target of 5en% from free sugars. This would be equivalent to ~25 g/day for women and ~35 g/day for men within a typical 8 MJ and 11 MJ diet respectively. The suggested limits apply to sugars naturally present in honey, syrups, fruit juices and fruit concentrates as well as the more publicised commercially added sugars [29].

## 2. Solid Food *vs.* Liquid Beverage: Consequences for Energy Intake

There is a growing literature to support the belief that food rheology and food matrix effects play a significant role in satiety responses to all of the macronutrients, and a systematic review of short-term preload studies has recently shown energy compensation to be maximised when foods are presented in a semisolid or solid form [30]. Alcohol and available carbohydrates (CHO) [31] (particularly simple sugars) are the current primary concern, and for a number of years it has been proposed that it is the vehicle itself, *i.e.*, the liquid format of the nutrient intake, rather than the micronutrient composition *per se* that has the greatest influence on intake [31,32,33]. There is some evidence that we may regulate badly, at least in the short-term, when faced with “liquid energy/calories” such that energy-containing beverages may evoke weaker appetite and compensatory dietary responses than solid food [30,31,33,34,35]. Hence they may increase total intake when added freely (“*ad libitum*”) into a typical diet [36,37]. Since ultimately it is a positive energy balance that leads to weight gain, any food or beverage such as SSBs that may promote daily intake above expenditure may expose the individual to a greater risk of weight gain. The weaker compensatory response has been proposed to be a consequence of a number of important differences between solid foods and beverages, including mouth feel and palatability amongst other orosensory responses, requirement for chewing, and total time required for consumption. For example, consuming 1 MJ of oranges as fruit (~4 oranges) takes 4–5 times as long as drinking an isoenergetic quantity of pressed fruit juice (~800 mL juice). Whether a beverage can induce a level of satiety comparable with an energy- and macronutrient-matched solid food is unlikely, and the incomplete compensation for energy consumed within the beverage may make these food formats a poor choice for individuals concerned with their daily energy intake and body weight.

Macronutrient specific effects on energy intake, *i.e.*, the satiety hierarchy of alcohol<fat <CHO<protein, may remain important despite these issues of food rheology. For example, there is evidence of enhanced appetite responses when volume matched isoenergetic mixed macronutrient (protein/fat/CHO) [38] or high protein [39] drinks replace CHO-only sugary beverages. Protein beverages are commonly of dairy whey or casein origin, taking the format of “thick shakes” rather than the clear water-based, high CHO SSBs and it is this difference in beverage format which may be driving the suppression of energy intake. Whilst low dose (up to 20 g per 500 mL, 4% w/w) protein-enriched water beverages can be formulated, and there is some evidence that they can promote suppression of hunger relative to a taste matched water beverage [40], there is little evidence of energy compensation for these protein beverages and hence may also result in the passive overconsumption as seen for both ABs and SSBs. A caveat for many of these studies however is their short duration. Far fewer long-term studies have been conducted which compare liquid *vs.* solid format foods. An isoenergetic, 4-week intervention in 15 adults supplemented with ~2 MJ/day of a liquid (SSB) or solid (jelly beans) CHO format showed good compensation during the day for the jelly beans but very poor or no compensation for the additional energy when given as a beverage [41]. In addition body weight and body mass index (BMI) increased significantly during the SSB phase of the study. In a more recent, longer term, intervention 34 lean and overweight adults were given isoenergetic beverage or solid forms of fruits and vegetables for two months [42]. There were notable differences in response between the participant groups with the lean group showing much better dietary compensation and no significant weight gain on the solid arm whilst poor compensation in the overweight group led to significant weight gain. Again the beverage arm led to poor compensation in the overweight but also in the lean group who had responded well when given the energy matched solid foods. A follow up publication by this research group also showed that appetitive responses assessed immediately after the meals were weaker following consumption of the beverages [43].

## 3. Energy Intake and Body Weight

### 3.1. Alcoholic Beverages (ABs)

The role of ABs and body weight has long been controversial. Alcohol intake is ubiquitous in adults in many countries globally, with levels of intake varying. In the 2006 Health Survey for England in a cohort of 8864 adults mean alcohol consumption was reported as 27en% and 19en% of recommended total daily energy intake in men and women respectively on the heaviest drinking day of the dietary report, and there was a positive association between alcohol energy and obesity [44]. There are a number of reasons that ABs have been implicated in weight gain and obesity. Alcohol (ethanol) contains more energy per gram (29 kJ/g) than either protein (17 kJ/g) or CHO (16 kJ/g), lying second in the hierarchy behind energy dense fat (37 kJ/g). The total energy content of a beverage of course varies considerably depending upon the format, which is typically separated into three groups comprising fermented beers and ciders, fermented wine, and distilled spirits where ethanol produced by means of fermenting grain or fruits is concentrated using a distilling process. In turn the content of alcohol by volume (ABV) varies widely between ~3%–40% [7], as does the total energy content. For example, the energy content of a typical glass of beer, wine or whiskey is 600 kJ, 360 kJ and 330 kJ, each also with varying content of CHO which contributes to the energy derived from ethanol within the beverage as consumed. When the beverage is consumed, the rapid absorption of ethanol into various body tissues means that even moderate consumption may affect the individual. Since portal circulation from the gut passes first through the liver, the majority ingested is metabolized within the liver, with obligatory oxidation beginning shortly after consumption of the drink. There is no reservoir within the body for alcohol storage and since urinary and respiratory excretion of excess alcohol represent only a minor route of disposal, it must rapidly increase alcohol oxidation following consumption. Alcohol metabolism involves at least three distinct enzymatic systems including alcohol dehydrogenase (ADH), microsomal ethanol-oxidising system (MEOS), and a non-oxidative pathway catalysed by fatty acid ethyl ester (FAEE) synthase. Oxidation of alcohol can also occur via the activity of catalase, but this plays a more minor role in alcohol metabolism under normal physiology. Adverse physiological effects are primarily due to the increased level of metabolites NADH and acetaldehyde rather than increased levels of ethanol *per se*.

#### 3.1.1. Short-Term Intervention Studies

**Figure 1:**
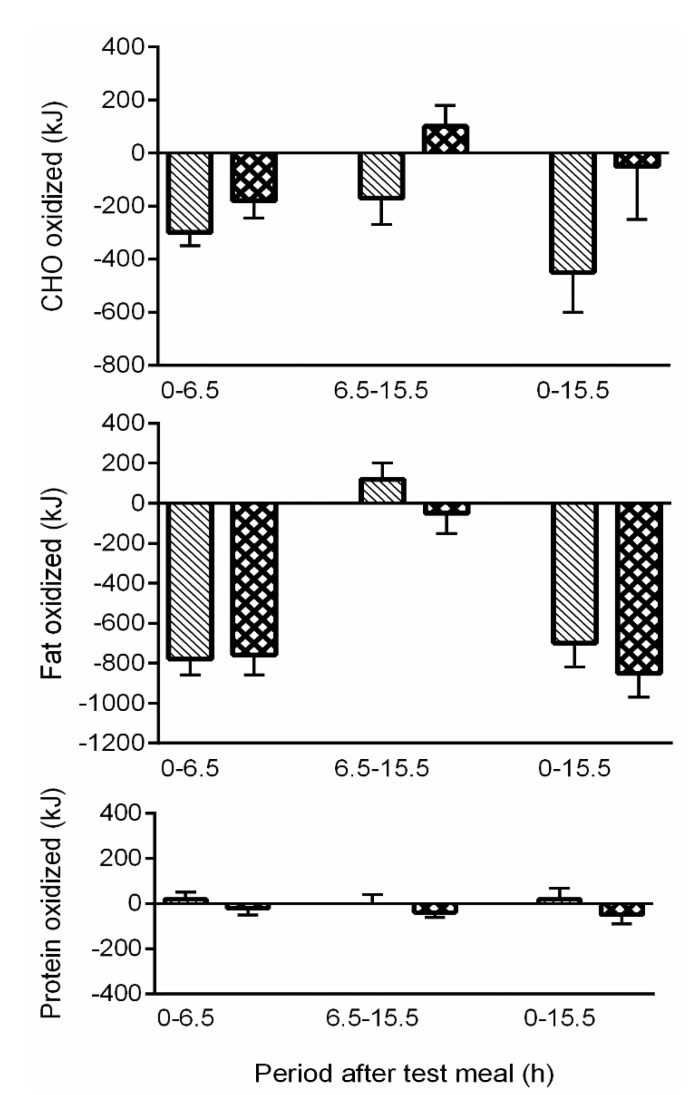
Change in macronutrient oxidation over 15.5 h in response to addition (ADD) or substitution (SUB) of alcohol into a test meal, relative to a no alcohol control. SUB (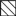), 50en% from CHO substituted for alcohol; ADD (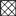), 50en% from CHO added as alcohol. Both addition and substitution of alcohol within a meal has an immediate suppressive effect on CHO and fat oxidation (from Murgatroyd *et al.*, 1996 [46], with permission).

This requirement for the obligatory oxidation of alcohol has long been known to have implications for other food macronutrients consumed at the same eating/drinking occasion, of particular consequence being the suppression of the rate of oxidation of both fat and CHO which leads in turn to increased storage of these substrates [45,46] (see Figure 1), and hence changes in body weight and composition. Metabolic evidence therefore supports the premise that an increase in alcohol intake will suppress oxidation and increase the storage and deposition of body fat, a key factor in weight gain and increased adiposity [47].

In 2010 Yeomans reviewed the role of alcohol on appetite and energy balance, and concluded from short-term clinical studies that energy consumed as alcohol was additive to that from other dietary macronutrient food and beverage sources, and hence led to short-term passive over-consumption of energy [48]. Specifically, that alcohol consumed before or with a meal tended to increase food intake, the so called “aperitif effect”, probably through enhancing the short-term reward effects of food. Certainly short-term preload intervention studies, including early work from my laboratory where consumption of ~1 MJ alcoholic beverage went unrecognised [36], have commonly shown no compensatory decrease in energy intake at a later meal, a greater total energy intake and hence propensity to enhance weight gain. The absence of longerterm RCTs, the conduct of which is precluded by the established adverse effects of prolonged alcohol consumption [49], has prevented cause and effect between intake of ABs and weight gain being established. In turn this has funneled considerable emphasis on to the body of observational data, despite the likelihood of bias as a consequence of alcohol under-reporting.

#### 3.1.2. Observational Studies

Contrary to the short-term experimental evidence of a plausible causative relationship between increased alcohol intake driving increased adiposity, is a long standing body of epidemiological data. Observational studies have shown a positive, negative, or no relationship between alcohol intake and body weight [3,50,51]. The lack of consensus with the observational data may be due to the difficulty of assessing alcohol intake, in the absence of independent biomarkers, an issue which remains unresolved with a significant problem of under-reporting within epidemiology [52]. A review by Suter [50] concluded that despite the difficulty in assessing alcohol intake as well as controlling for different confounders of the energy intake, expenditure and balance equation, the conflicting epidemiologic data could be explained in most instances, and that alcohol calories *do* count particularly in combination with a high-fat diet and in those concerned with overweight and weight loss.

Nevertheless, a recent systematic review of large cross-sectional and long-term prospective cohort studies again has concluded no conclusive evidence of a positive association between alcohol consumption and weight gain [51]. The authors reported that these data were inconclusive on the relationship between alcohol intake, body weight and adiposity, and made similar conclusions for the body of evidence of shorter experimental trials. They reported that in light-moderate “social” drinkers there was some evidence that consumption of distilled spirits was positively associated with weight gain, whilst perhaps surprisingly wine was associated with protection against weight gain. The adverse relationship between alcohol intake and weight gain was more commonly found in studies reporting populations with higher levels of alcohol consumption, “heavy” drinkers, raising the suggestion that it is primarily only in heavy alcohol consumers that weight gain and obesity is a significant risk [51]. Similar conclusions have been reached in a 2015 review [3] commenting that alcohol consumption has probably contributed to the excess energy intake associated with weight gain in some individuals, but that available evidence is conflicting and precludes strong conclusions on the effect of ABs on obesity risk.

The body of observational data showing no association between alcohol intake and increased body weight led to the question “do alcohol calories count?” [53] and the theory of the ethanol futile cycle as a possible pathway of energy wastage to explain the absence of a positive relationship. As ethanol is converted to acetaldehyde through the MEOS pathway and simultaneously from acetaldehyde to ethanol via the ADH pathway, a potentially “futile” cycle is set up which would represent a net loss of 6 ATP per cycle. No experimental evidence in support of this theory has been generated however, with NMR spectroscopy used to test blood collects after administration of H-labelled ethanol but with no evidence of futile cycling detected [54]. Clearly, scientific consensus in this area is still to be reached and may require better reporting methods and independent validation of alcohol intake data to provide a definitive answer.

### 3.2. Sugar Sweetened Beverages (SSBs)

**Figure 2:**
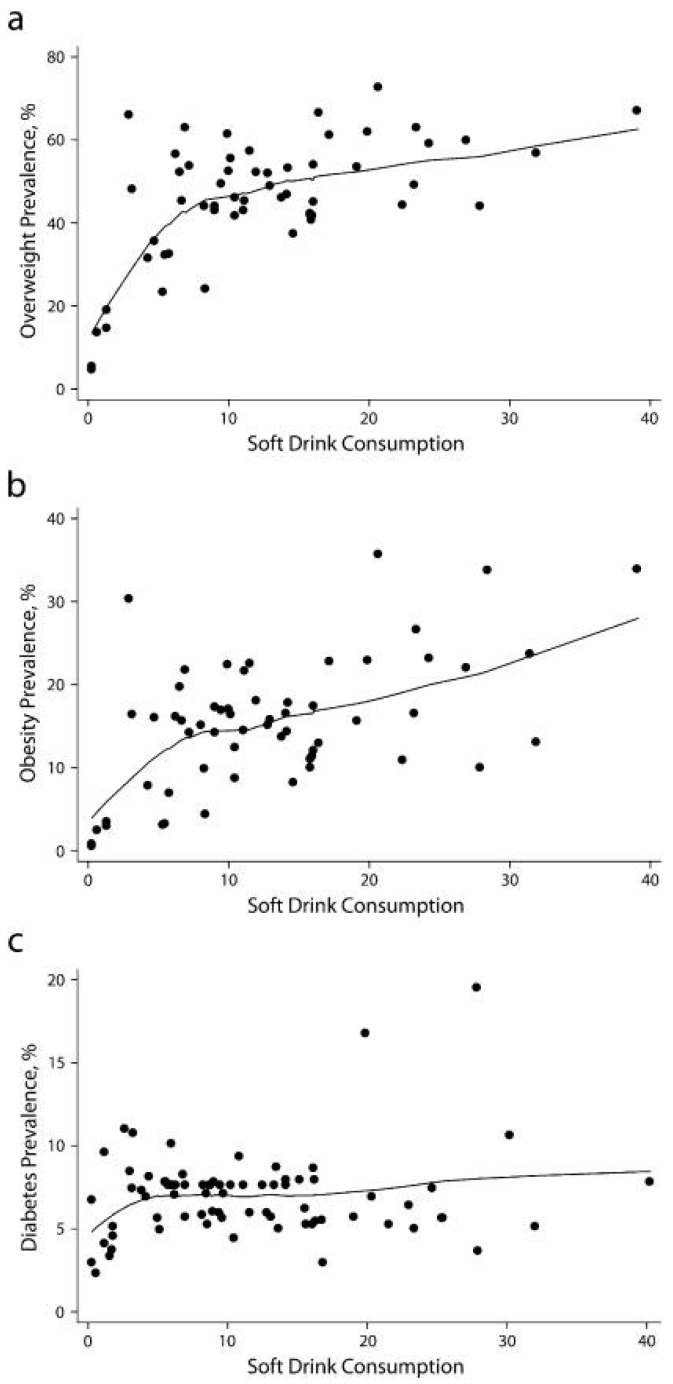
Relationship of soft drink consumption to the prevalence of (**a**) overweight, (**b**) obesity, (**c**) type 2 diabetes in adults from 75 countries, based on data from EuroMonitor Passport Global Market Information Database on beverage intake and World Health Organization’s Global Database on overweight and obesity (from Basu *et al*, 2013 [24], with permission).

As noted previously, SSB consumption continues to rise globally. Basu *et al.*’s recent 75 country analysis showed that each 1% rise in soft drink consumption was associated with an additional 4.8 overweight adults per 100 (Figure 2).

Of course, observational data such as this can provide evidence only of an association and says nothing of causation. Greater prevalence of overweight and obesity however is supported by data showing that, whilst the energy content of a food is clearly an important driver of satiety, consumption of liquid CHO may be poorly recognised and engender less compensation than an isoenergetic high-CHO solid format meal. This has been shown in several [31,55], although not all, studies [56]. How strong is this evidence [57], and if there is a causative relationship with SSBs, whether this may be a consequence of the sweet nature of sugary CHO beverages, the high energy content, or simply the food form remains under debate [31]. What is clear, however, is that any dietary item that promotes a sufficiently high energy intake to distort energy balance, whether as a consequence of high palatability and consumer desire for consumption or an absence of compensatory response to energy within the beverage, may be of greater risk of inducing weight gain. It has long been proposed that consumption of highly palatable food items, including those rich in sugar such as the SSBs, is overly stimulating to the brain’s reward pathways [58] and may serve as a reinforcer, in turn increasing the likelihood of the behavior reoccurring.

#### 3.2.1. Observational Studies

Malik and colleagues [4,59] recently reviewed the epidemiological evidence including 22 cohort studies, of which 7 were in adult populations and 15 in children [4]. They concluded that one additional serve of SSBs was associated with a 0.05–0.06 unit increase in BMI in children and 0.12–0.22 kg additional weight gain in adults, as assessed over a one year period. So, whilst not all observational studies have found a clear association between SSB intake and body weight [60,61], there is a growing body of evidence for those that do to show a positive association. Variability in study design, population size, and duration or absence of follow up all contribute to challenges in interpreting these data sets, however [62]. Notable in the absence of a relationship has been the large NHANES data set in more than 38,000 US adults aged 20–74 years, where no significantly association between obesity risk and SSB consumption pattern has been found, such that those who reported frequently consuming SSBs did not have either a higher BMI or greater obesity rates than those who were self-reported infrequent consumers [60]. A recent meta-analysis conducted to evaluate the strength of evidence for an independent relationship between SSBs and the risk of obesity when adjustment for energy intake was made, has further contributed to the debate, having concluded that the data in adult, adolescent and child cohorts fail to provide consistent evidence [63]. The authors argued that it is not clear how SSBs contribute to EI and obesity in a manner different to other food groups previously identified as making a greater energy contribution than these nutritive beverages. In the US, for example, this includes grain based desserts, yeast breads and chicken-containing foods [64].

#### 3.2.2. Intervention Studies

Intervention data has tended to support a causal relationship between increased intake of SSBs and weight gain, but again the strength of evidence has been questioned [57]. Previously Mattes and colleagues confirmed water based, energy-containing beverages increased the risk of a positive energy balance [32], and adding these beverages to a meal has been shown to promote energy intake at that meal [37,65]. Mattes in turn has conducted a meta-analysis of RCTs, including those which had intervened over a period of three weeks or more [66], in which it was shown that the addition of SSBs in the form of soft drinks, fruit juice beverages and chocolate milks caused a dose-dependent increase in weight gain. Removing SSBs from the diet of overweight individuals was shown to prevent further weight gain and/or promote greater weight loss than their lean counterparts [66]. Systematic review of 10 RCTs in adults and children by Malik *et al.*, also supported the conclusion of a positive causal relationship [4]. Mechanisms underpinning overconsumption of energy when consumed in a liquid format have been proposed. Tucker and Mattes reviewed these mechanisms in detail [67], summarising the systems effected as 1 or more of cognitive, oral processing, gastric emptying, gastrointestinal (GI) transit and endocrine response. It had been argued that the combination of long term follow up in observational studies and shorter term RCTs had resolved the controversy, and confirmed a causal relationship [68], however, the debate has been reignited in a recent update of the Mattes meta-analysis [66] conducted by Kaiser and colleagues [57]. Inclusion of recent trials and updated analysis found RCT evidence to be equivocal, particularly with respect to strength of evidence in support of decreased SSB consumption reducing the prevalence of obesity. Perhaps surprisingly, they showed that energy compensation in response to intake of SSBs may be as high as 85% when compared to a theoretical model of weight gain assuming no activity or dietary changes during SSB supplementation. In this update the authors report “*added SSBs to persons’ diet showed dose-dependent increases in weight*”, but conversely, “*attempting to reduce SSB consumption showed an equivocal effect on body weight/composition indices*”.

## 4. Children and Adolescents

Most studies report that consumption of SSBs among children and adolescents is continuing to increase [17,69,70], and in turn considered to be a particularly at risk group for development of overweight and obesity [21,71] and an important target for prevention. Growing evidence of adverse effects on body weight has led to a number of public health initiatives in the US, including both removing soft drinks and sweet flavoured milks from schools [72] and increasing government taxes on these products, although notably the outcome from such initiatives to date have been mixed [73,74]. Mexico, the country which tops the OECD country listing for adult obesity prevalence alongside the US and New Zealand with ~1 in 3 adults obese [75], have also responded through government-led initiatives having instituted a 10% excise tax for SSBs at the beginning of 2014 [76]. National government initiatives have also been proposed within Europe, including the UK [77,78]. Direct comparison between studies conducted in children and adolescents are somewhat hindered by variable methods used to both classify and assess SSB intake as well as to define body composition and overweight (BMI *vs.* skinfolds) between countries such as the US and some European countries. The weight of evidence in Europe appears lower than the US with not all studies [79,80] showing an association between SSB intake and overweight, although many do [71,81] in agreement with a growing literature of increased SSBs, energy intake, overweight, and obesity in children exposed to a typical western diet pattern [61,62,82,83]. As observed for the data in adults, however, there remains controversy as to the strength of evidence and demonstration of causation in children [57], not least due to methodological issues with trials including few RCTs and poor compliance to intervention [28]. Evidence from the RCTs which typically comprised advice to decrease SSBs, alongside other free sugars, has failed to show change in body weight [28]. In turn, debate continues as to whether high-CHO energy drinks should be banned for children under 16 years of age [84], in order to promote adherence to the recent WHO recommendations to target <5en% from free sugars.

### 4.1. Preschool Children

**Figure 3:**
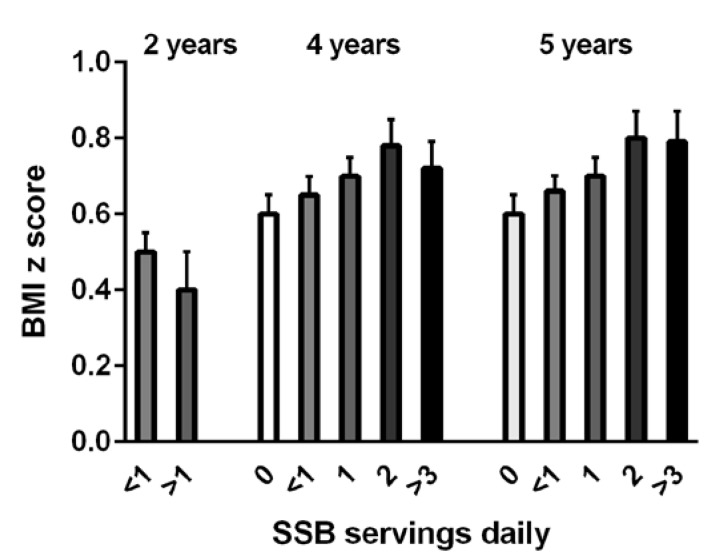
Mean BMI z scores of children aged 2, 4, and 5 years showing daily consumption of sugar sweetened beverages (SSBs), which was significantly different *versus* nondrinkers of SSBs (from de Boer *et al.*, 2013 [85], with permission).

Whilst there is less published data available in younger preschool children, the American Academy of Pediatrics has recommended that young children refrain from intake of SSBs [62]. A recent analysis of the US Early Childhood Longitudinal Survey Birth Cohort conducted in almost 1000 young children showed a significant relationship of SSBs with overweight and obesity. Consumption was associated with both higher BMI-z scores and higher weight status in these young preschool children, aged 2–5 years [85], (see Figure 3).

A number of other studies have also reported an association between SSB consumption and higher weight status in young children [82,86,87,88,89,90]. Not all studies in this younger age group have been supportive of an association however [61,91,92,93,94], including two studies which were highlighted in a review by De Boer and colleagues [85] to be financed in part by the soft drinks industry [61,91]. Some of those reporting no effect have also been criticised [85] as reporting only cross-sectional data [61,88,89,95], studies derived from regional populations [82,90], and those with small sample size [86,87,92,93,94]. In the comprehensive meta-analysis by Te Morenga and colleagues no RCTs were identified in preschool children [28].

### 4.2. Milk as a Beverage

Liquid milk, with its significant lactose content, has also come under scrutiny, although the relationship between milk consumption and BMI in children is far more mixed than that for water based SSBs [91,96,97,98,99]. A review of the US NHANES data in young children aged 1–5 years over the last 30 years has shown an inverse relationship between liquid milk and SSB intake, with milk decreasing and fruit juice/SSBs increasing [100]. Some national guidelines in countries such as the US do retain focus on dairy during childhood, but the issue has been of lipid rather than CHO content, and guidelines promote consumption of low energy dense low-fat and/or fat-free milk beverages [101]. Skim milk has been shown to suppress short term appetite when compared with isoenergetic fruit juice in adults [102], and also shown in children [103]. Modelling of NHANES data in a total of 8112 children aged 2–19 years has shown that replacing whole, reduced-fat and flavoured milk with low-fat or skimmed milk generated a predicted reduction of between 1.4%–2.5% of total energy intake, and hence interpreted by the authors as having potential to reduce energy as well as total and saturated fat intake [104].

## 5. Are Beverages Tipping the Balance?

There is little doubt that intake of energy within a liquid format has increased within westernized diets over the past 20 years, that the two main macronutrient sources are alcohol and mono/disaccharide CHOs, and that this now represents a large proportion of total daily energy intake in many individuals. Much of the controversy surrounding the role of alcohol and adiposity stems from a lack or absence of interpretable clinical trial data. The inability to conduct RCTs to determine a causative relationship between ABs and body weight is a major barrier. In turn, observational studies have been hampered by the challenge of macronutrient specific under-reporting and the absence of independent validation methods to verify habitual alcohol intake. These issues, which are long associated with reporting of food macronutrients are likely worse for alcohol reporting as an item less central to a meal [105]. Unsurprisingly, as a consequence, epidemiology has been largely inconclusive with respect to the relationship between alcohol intake and body weight, leading researchers down blind alleys searching for energy wastage cycles to explain studies which have reported no association. Scientific consensus will require better reporting methods and independent validation of alcohol consumption data in order for the large observational studies to provide a robust answer. It is possible that there are important differences between light, moderate and heavy alcohol consumers and their risk of overweight and obesity, but again associations can only be adequately investigated should validated biomarkers become available for large population studies.

Observational outcomes may be clearer for SSBs where the body of evidence tends to support an association between intake of CHO beverages and higher body weight [68], although it is not a universal finding. Variation both in protocol design and subsequent selection of clinical studies for systematic review or meta-analysis has been proposed as an underpinning issue here [63], preventing consensus. Further controversy lies in establishing causality in the development of overweight and obesity in either adults or children [57]. Longer-term RCTs which have introduced or removed SSBs within the diet tend to support this causative relationship with weight gain, but certainly not all studies do [57]. In order to avoid a significant positive effect on body weight and gradual adiposity “creep”, additional energy consumed as liquid format must be compensated for by a parallel decrease in the food macronutrients. Short-term studies suggest that this is unlikely, with poor short-term regulation of “liquid calories” and failure of energy-containing liquids to evoke a strong satiety response. There is some evidence in support of Mattes premise that the menace may indeed be the medium [31], and meta-analysis of RCTs supports a dose-dependent increase in body weight gain, albeit far less than would be predicted if energy from SSBs elicited no compensation within other components of the diet [57]. Clearly, there is some compensation for this liquid energy, although it does appear that beverages may be tipping the body weight balance in favour of weight gain, with SSBs and possibly also ABs playing a role. Removal of energy-containing beverages from our diet may be an appropriate public health message to support those interested in preventing weight gain as well as those focused on weight loss, and in the face of the expanding global waistline arguably may be an appropriate step to take ahead of unequivocal evidence. Whether this can be achieved at the level of the individual with no change in the ready availability of these beverages is not known, but it appears likely that regulatory and national government intervention may be required to achieve significant and prolonged environment change in order to tip the balance in favour of better body weight control.
